# Short-tunnel submucosal tunneling endoscopic resection for the removal of a rectal gastrointestinal stromal tumor above the dentate line

**DOI:** 10.1055/a-2686-2775

**Published:** 2025-09-19

**Authors:** Xingbin Ma, Huaiyuan Ma, Qiong Niu

**Affiliations:** 1 562131 Department of Gastroenterology and Hepatology, Binzhou Medical University Hospital, Binzhou, China; 2 562131 Endoscopy Center, Binzhou Medical University Hospital, Binzhou, China

The management of rectal gastrointestinal stromal tumors (GISTs) is complex, particularly for small lesions located in functional areas near the dentate line 1 . This report describes a case of short-tunnel submucosal tunneling endoscopic resection (STER) 2 for the removal of a rectal GIST near the dentate line, aiming to expand clinical insights into minimally invasive approaches.

A 72-year-old woman undergoing colonoscopy for diarrhea was found to have a hemispherical lesion (0.6 cm) on the anterior wall of the distal rectum, 2–3 cm above the dentate line on the anterior wall of the distal rectum ( Fig. 1 **a** ). Endoscopic ultrasound revealed a lesion measuring 6.4 mm × 3.5 mm, originating from the muscularis propria ( Fig. 1 **b** ).

**Fig. 1:**
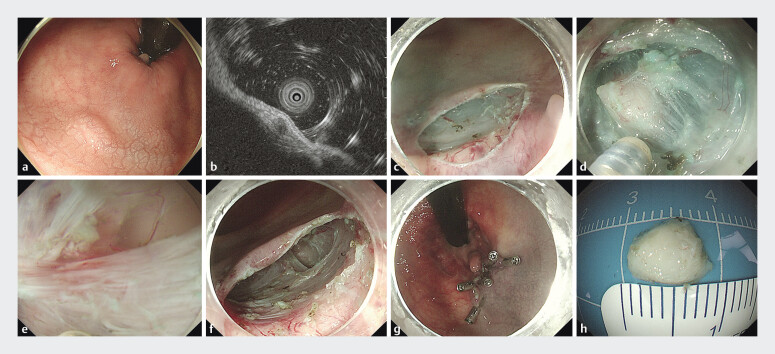
Endoscopic resection of rectal GIST via short-tunnel STER. **a** Endoscopic view of a rectal tumor located 2–3 cm above the dentate line. **b** Endoscopic ultrasound demonstrates the lesion originated from the muscularis propria. **c** Transverse mucosal incision at the dentate line for tunnel entry creation. **d** Exposure of the muscularis propria tumor after submucosal dissection. **e** Underwater-assisted tumor flotation enhances visualization for en bloc resection. **f** Short-tunnel STER. **g** Titanium clip closure ensures mucosal integrity. **h** Resected tumor specimen with intact pseudo capsule.

The patient underwent STER under propofol sedation with CO _2_ insufflation, using a standard gastroscope (GIF-H290T) equipped with a transparent cap (D-201-11804) (Olympus, Tokyo, Japan), VDK-KM-15-195-D knife 1.5 mm (Vedkang, Jiangsu, China). After marking the tumor with methylene blue, a mucosal incision knife was used to create the tunnel entrance above the dentate line ( Fig. 1 **c** ). The lesion was identified at the muscularis propria during the procedure, and the underwater technique was employed to float and successfully excise the tumor. The procedure lasted approximately 20 minutes ( Fig. 1 **d–h** , Video 1 ).

Short-tunnel submucosal tunneling endoscopic resection for removal of rectal gastrointestinal stromal tumor above the dentate line.Video 1

Postoperative pathological analysis confirmed a rectal GIST with a mitotic index of <5/50 high-power fields, positive staining for CD117, DOG-1, CD34, and SDHB, and a Ki67 index of <1% ( Fig. 2 ). The tumor was classified as very low-risk, and no complications occurred. The patient was advised to undergo regular surveillance.

**Fig. 2:**
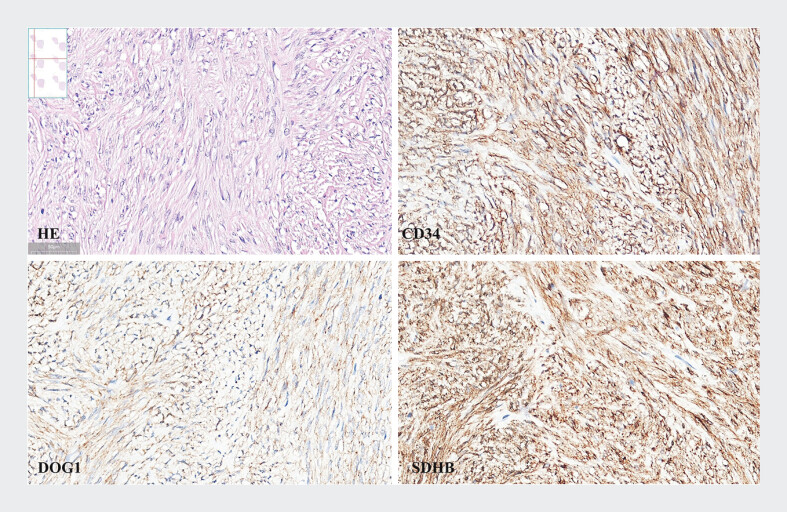
Pathological and immunohistochemical features of GIST.

Based on our experience, the short-tunnel technique offers simplified manipulation and predictable outcomes. Underwater dissection minimizes mucosal injury and enhances visualization during the procedure 3 . Besides, in patients with internal hemorrhoids, further experience is needed before making definitive recommendations.

Endoscopy_UCTN_Code_TTT_1AQ_2AD_3AZ

## Notes

Endoscopy E-Videos https://eref.thieme.de/e-videos
*E-Videos* is an open access online section of the journal *Endoscopy* , reporting on interesting cases and new techniques in gastroenterological endoscopy. All papers include a high-quality video and are published with a Creative Commons CC-BY license. Endoscopy E-Videos qualify for HINARI discounts and waivers and eligibility is automatically checked during the submission process. We grant 100% waivers to articles whose corresponding authors are based in Group A countries and 50% waivers to those who are based in Group B countries as classified by Research4Life (see: https://www.research4life.org/access/eligibility/ ). This section has its own submission website at https://mc.manuscriptcentral.com/e-videos .
